# In silico prediction of polyketide biosynthetic gene clusters in the genomes of *Hypericum*-borne endophytic fungi

**DOI:** 10.1186/s12864-024-10475-z

**Published:** 2024-06-03

**Authors:** Linda Petijová, Jana Henzelyová, Júlia Kuncová, Martina Matoušková, Eva Čellárová

**Affiliations:** Department of Genetics, Institute of Biology and Ecology, Faculty of Science, Pavol Jozef Šafárik University in Košice, Mánesova 23, Košice, 04154 Slovakia

**Keywords:** Hypericum, Anthraquinones, Endophytic fungi, Biosynthetic genes, Bioinformatic analysis

## Abstract

**Background:**

The search for new bioactive natural compounds with anticancer activity is still of great importance. Even though their potential for diagnostics and treatment of cancer has already been proved, the availability is still limited. Hypericin, a naphthodianthrone isolated essentially from plant source *Hypericum perforatum* L. along with other related anthraquinones and bisanthraquinones belongs to this group of compounds. Although it has been proven that hypericin is synthesized by the polyketide pathway in plants, none of the candidate genes coding for key enzymes has been experimentally validated yet. Despite the rare occurrence of anthraquinones in plants, their presence in microorganisms, including endophytic fungi, is quite common. Unlike plants, several biosynthetic genes grouped into clusters (BGCs) in fungal endophytes have already been characterized.

**Results:**

The aim of this work was to predict, identify and characterize the anthraquinone BGCs in de novo assembled and functionally annotated genomes of selected endophytic fungal isolates (*Fusarium oxysporum, Plectosphaerella cucumerina, Scedosporium apiospermum, Diaporthe eres, Canariomyces subthermophilus*) obtained from different tissues of *Hypericum* spp. The number of predicted type I polyketide synthase (PKS) BGCs in the studied genomes varied. The non-reducing type I PKS lacking thioesterase domain and adjacent discrete gene encoding protein with product release function were identified only in the genomes of *C. subthermophilus* and *D.* *eres*. A candidate bisanthraquinone BGC was predicted in *C.* *subthermophilus* genome and comprised genes coding the enzymes that catalyze formation of the basic anthraquinone skeleton (PKS, metallo-beta-lactamase, decarboxylase, anthrone oxygenase), putative dimerization enzyme (cytochrome P450 monooxygenase), other tailoring enzymes (oxidoreductase, dehydrogenase/reductase), and non-catalytic proteins (fungal transcription factor, transporter protein).

**Conclusions:**

The results provide an insight into genetic background of anthraquinone biosynthesis in *Hypericum*-borne endophytes. The predicted bisanthraquinone gene cluster represents a basis for functional validation of the candidate biosynthetic genes in a simple eukaryotic system as a prospective biotechnological alternative for production of hypericin and related bioactive anthraquinones.

**Supplementary Information:**

The online version contains supplementary material available at 10.1186/s12864-024-10475-z.

## Background

Medicinal plants are important producers of various bioactive secondary metabolites (SM) with high impact for the pharmaceutical industry. While some groups of SM are widespread in the plant kingdom, the other, even with high therapeutic potential are restricted to certain plant taxons. In some cases, the total content of biosynthesized SM is a result of a synergic crosstalk between the plant and endophytic microorganisms inhabiting the host tissues. Endophytic fungi reside inside the plant tissues without causing a disease to their host plant. As many endophytes are able to produce in vitro the same compounds as their hosts, these fungi are assumed as sustainable cell-factories for heterologous production of diverse bioactive metabolites [1].

SMs that are widespread in microorganisms and rarely present in plants include anthraquinones. Natural anthraquinones are bioactive compounds synthesized predominantly via polyketide pathway. In higher plants, there are two main proposed routes leading to anthraquinones: the polyketide pathway by type III polyketide synthase and the shikimate or chorismate/O-succinyl benzoic acid pathway (reviewed by Mund and Čellárová [2]). The representatives of the genus *Hypericum* belong to few plant taxons that are important producers of (bis)anthraquinones, and are the only plant producers of hypericin and its derivatives. Hypericin and skyrin are promising agents in the anticancer therapy [3, 4]. Despite the biosynthesis of hypericin has not been fully elucidated, emodin and/or emodin anthrone are considered as possible precursors. Based on the results of LC–MS analyses, Kimáková et al. [5] proposed hypericin biosynthetic route via bisanthraquinone skyrin. Later Rizzo et al. [6] identified atrochrysone as a putative precursor and skyrin as a side product of hypericin biosynthesis.

Up to date, dozens of endophytes were isolated from *Hypericum* plants of different provenance [7–9]. Most of the identified isolates are filamentous ascomycetes (e.g. *Fusarium, Diaporthe, Alternaria, Septoria*), less frequent basidiomycetes (*Schizophyllum, Phlebia*), and mucoromycetes (*Mortierella*) (Fig. S1). According to the metabolomic analyses, axenic cultures of several isolates were able to produce small quantities of the host plant-derived anthraquinones, especially hypericin, pseudohypericin, emodin and skyrin during the first subcultures. However, the genetic and epigenetic background of anthraquinone biosynthesis in *Hypericum*-borne endophytic fungi has not been studied yet. The knowledge on pathway and regulation of anthraquinone biosynthesis in fungal endophytes may significantly contribute to the understanding of hypericin biosynthesis in *Hypericum* spp. [2].

Fungal anthraquinone biosynthetic gene clusters (BGCs) usually consist of genes encoding enzymes catalyzing biosynthetic steps leading to basic anthraquinone skeleton, other enzymes tailoring the final product, transport and regulation genes. High-throughput genome sequencing and bioinformatics are powerful approaches that markedly help to discover novel putative cryptic BGCs, prior to further metabolomic analyses and functional validation of candidate biosynthetic genes. Moreover, most of the anthraquinone BGCs become transcriptionally inactive after several subcultures and whole-genome screening may contribute to decipher the hidden biosynthetic potential.

The objectives of the present study are:

1. In silico prediction and functional annotation of polyketide biosynthetic gene clusters in de novo assembled and annotated genomes of selected fungal endophytes isolated from *Hypericum* spp.
2. Comparative analysis of the predicted and already identified biosynthetic gene clusters of emodin-derived polyketides.

## Results

### General characteristics of the assembled genomes

The genomic DNA of fungal endophytic isolates *Fusarium oxysporum* 1LF1-1 and 8RF1-3*, Scedosporium apiospermum* 2RF1-5*, Plectosphaerella* *cucumerina* 2SF1-3, *Diaporthe* *eres* 17SF1-1, and *Canariomyces subthermophilus* CS-A were sequenced and assembled de novo with estimated coverage of 100X. The genome of *C. subthermophilus* was sequenced for the first time. The assembly statistics is summarized in Table 1. The genomes were assembled into scaffolds with the N50 value varying from 79,132 bp to 325,226 bp. The assembled genomes of *F.* *oxysporum* isolates 1LF1-1 and 8RF1-3 reached very similar statistics of scaffold lengths.

**Table 1:** Characterictics of de novo assembled genomes of *Hypericum*-borne endophytes

		***Canariomyces subthermophilus*** **CS-A**	***Fusarium oxysporum*** **1LF1-1**	***Fusarium oxysporum*** **8RF1-3**	***Diaporthe eres*** **17SF1-1**	***Plectosphaerella cucumerina*** **2SF1-3**	***Scedosporium apiospermum*** **2RF1-5**
**Assembly statistics**	**GC content (%)**	54.46	47.63	47.63	49.48	58.52	49.93
	**Assembly length (Mb)**	31.12	48.06	48.03	60.34	35.48	43.67
	**N50 (bp)**	138,935	313,603	325,226	118,005	234,631	79,132
	**L50**	70	45	43	149	50	171
	**# of scaffolds** **(> 0.1 Mb)**	114	122	124	188	125	107
	**Longest scaffold (Mb)**	0.68	1.39	1.42	0.47	0.77	0.39
**Predicted genes**	**tRNA**	178	324	325	168	352	271
	**rRNA**	37	60	58	34	76	49
	**Protein coding genes**	8,419	14,294	14,291	12,645	10,099	9,562
	**Predicted genes**	9,549	15,075	15,061	16,808	12,948	11,531
	**Mean gene length (bp)**	1,849	1,675	1,675	1,742	1,746	1,773
	**Mean exon length (bp)**	555	514	514	541	540	531
	**Mean intron length (bp)**	106	86	86	110	96	115
	**Mean number of introns per gene**	2.0	1.9	1.9	1.8	1.9	1.9
**Mitochondrial genomes**	**Size (kb)**	36.8	52.4	52.4	90.3	27.1	25.8
	**# of genes**	44	58	58	81	41	40
**Repetitive sequences**	**% of genome**	1.22	0.95	0.99	1.62	1.86	1.70
	**SINE**	35	34	34	38	56	27
	**LINE**	183	229	228	253	173	116
	**DNA elements**	52	53	57	53	50	25
	**LTR**	1	5	5	3	1	3
	**Simple repeats**	6,367	6,987	6,955	15,294	8,781	12,176
	**Repetitions predicted de novo**	114	79	87	65	42	42
**% of sequences aligned on reference**		59.11%^a^	84.90%	84.71%	97.39%	99.29%	99.58%

^a^Assembly was aligned on *Thermothielavioides terrestris* genome (related thermophilic fungus)

**Fig. 1:**
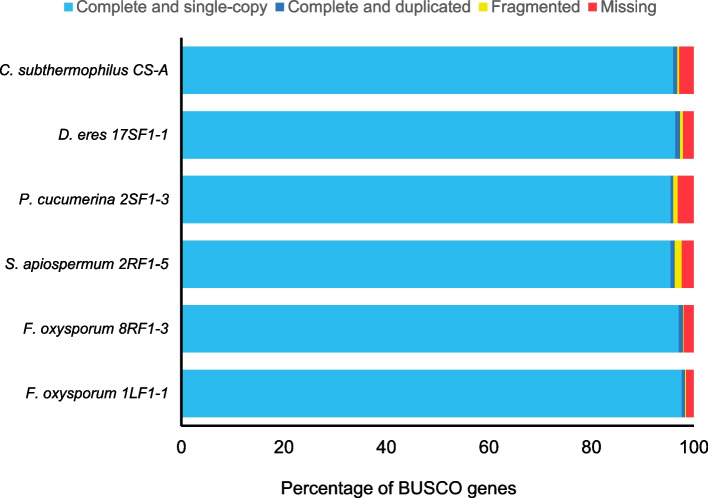
BUSCO analyses of studied fungal genomes

The assessment of assembly and annotation completeness confirmed that all the assembled genomes are appropriate for downstream analyses. Generally, more than 95% of the predicted genes were identified as complete and single-copy and the fraction of fragmented and missing genes did not exceed 4% (Fig. 1).

**Fig. 2:**
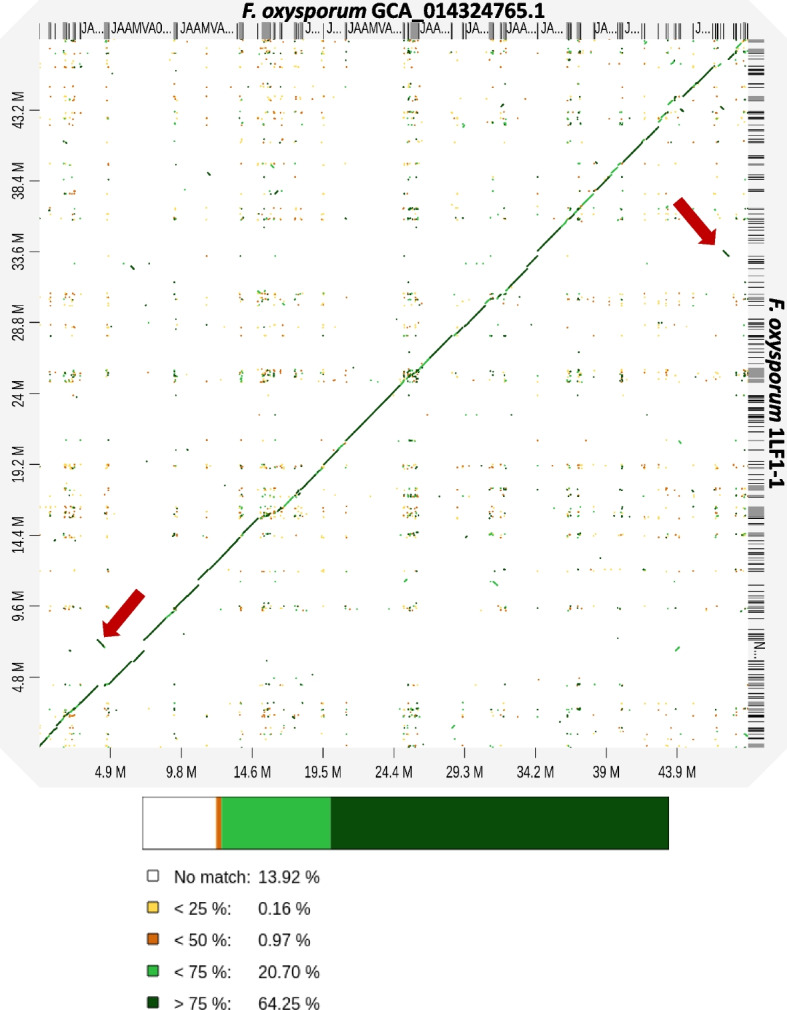
The representative alignment of *F. oxysporum* GCA_014324765.1 reference genome and *F. oxysporum* 1LF1-1. The observed translocation and inversion are marked by circles. The alignments are colored according to the sequence identity

The assembled genomes of *Hypericum*-borne endophytic isolates were compared to the assemblies obtained from NCBI database (endophytic fungus *F.* *oxysporum* GCA_014324765.1, plant pathogenic fungi *D. eres* GCA_022570805.1 and *P. cucumerina* GCA_014636675.1, soil fungus *S. apiospermum* GCA_002158515.1, and thermophilic fungus *Thermothielavioides terrestris* GCA_900343105.1). According to all-vs-all comparison of nucleotide sequences, 85 to 99.5% of the sequences were aligned to the reference genome assemblies. Occasional translocations and inversions were observed, especially in both aligned *F.* *oxysporum* genomes (Fig. 2, Fig. S2). The most remarkable deviation from diagonal line, indicating more frequent insertions or deletions, was found in *P.* *cucumerina* 2SF1-3 genome aligned to the genomic sequence of pathogenic *P.* *cucumerina* isolated from *Arabidopsis thaliana* (GCA_014636675.1) (Fig. S3)*.* The genomic rearrangements were less obvious in the aligned *D.* *eres* 17SF1-1 and *S.* *apiospermum* 2RF1-5, but the overall sequence similarity was relatively low (Fig. S4, Fig. S5). The genomic alignment of two distinct species *C. subthermophilus* CS-A and *T.* *terrestris* revealed remarkable differences with almost 60% contigs unaligned (Fig. S6).

The fungal mitochondrial genomes were assembled de novo from genomic data. The size of circular mitogenomes ranged from 26 to 90 kb (Table 1).

### Functional annotation and gene family clustering of genomic sequences

**Fig. 3:**
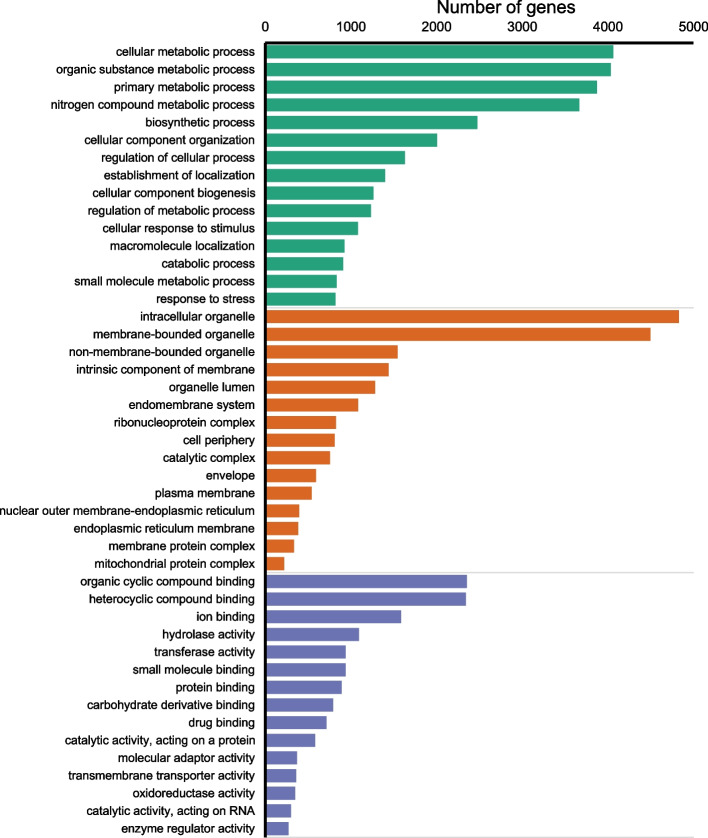
Gene ontology classification of *C. subthermophilus* genes. The major category Biological process is colored by green, Cellular component by tawny and Molecular function by Chetwode blue

**Fig. 4:**
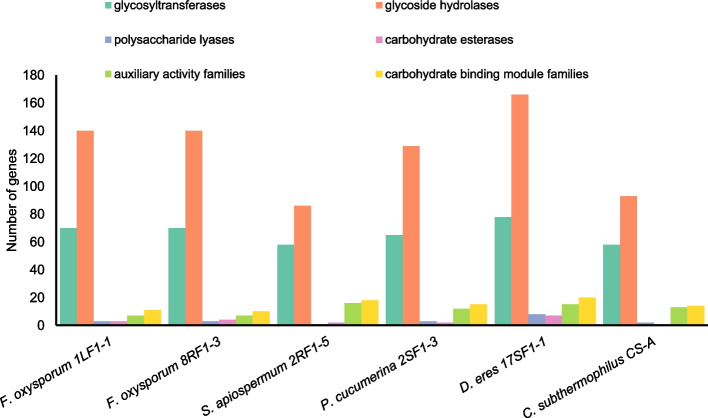
Distribution of carbohydrate-active enzymatic classes in the genomes of *Hypericum*-borne endophytic fungi

The protein-coding genes represented more than 75% of the predicted genes in all the studied genomes. Their putative functions were inferred by homology-based approach using more annotation sources. The hierarchical classification of GO terms showed that most of the predicted genes are associated with various biosynthetic processes, regulation of metabolism and stress response (Fig. 3). Majority of proteins with catalytic function showed aromatic compounds and ion binding activity. The whole-genome screening of carbohydrate-active enzymes (CAZy) revealed that glycoside hydrolases and glycosyltransferases were the most abundant in all studied genomes (Fig. 4). The lyases, esterases, and other auxiliary enzymes represented only minor part of enzymome.

Besides the prediction of protein-coding genes, the functional classification of identified genes was enriched by the prediction of selected non-coding RNAs. The numbers of tRNA and rRNA genes differed among the fungal isolates (Table 1). The whole-genome screening of repetitive regions revealed that transposable elements represented up to 2% of genome size.

The number of predicted genes in mitochondrial genomes varied from 40 (*S.* *apiospermum* 2RF1-5) to 81 (*D. eres* 17SF1-1) and comprised protein-coding genes, rRNA genes, tRNA genes, and open reading frames with unknown functions. Functional annotation of mitogenomes confirmed all 14 genetically conserved protein-coding genes associated with electron transport chain, except for missing *atp9* in *C.* *subthermophilus* CS-A. The gene encoding ATPase subunit 9 was found in the nuclear genomic sequence. Six additional genes encoding the potential mobile endonucleases were predicted in *D. eres* 17SF1-1 mitogenome. Several genes were duplicated, e.g. *cox1* gene encoding cytochrome oxidase subunit 1 were identified in *C. subthermophilus* CS-A (Fig. S7).

The clustering of protein-coding genes showed that at least 84% of genes were assigned to orthogroups in all the analyzed isolates (Fig. S8). The number of species-specific orthogroups varied among the studied genomes. The whole genome comparison of two *F. oxysporum* isolates did not reveal any species-specific orthogroup, but the genomes slightly differed in the number of genes unassigned to any orthogroup.

### Bioinformatic prediction of polyketide biosynthetic gene clusters

**Fig. 5:**
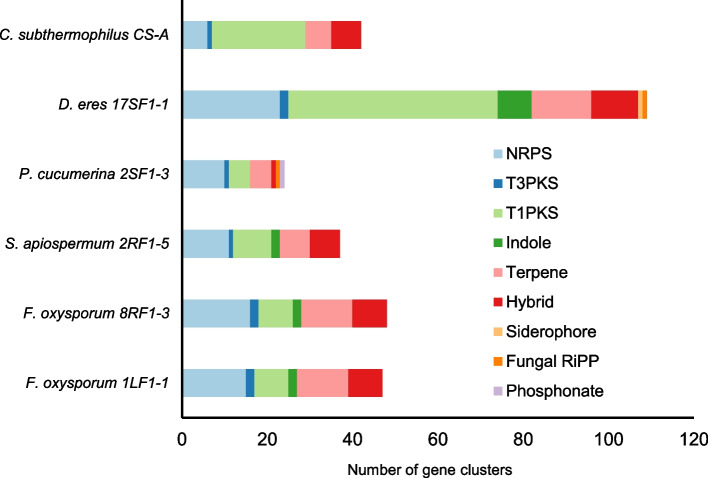
Predicted BGCs in six *Hypericum*-borne endophytic fungi. (NRPS – non-ribosomal peptide syznthase, T3PKS – type III polyketidsynthase, T1PKS – type I polyketidsynthase, fungal RiPP—ribosomally synthesized and post-translationally modified peptide)

The studied fungal endophytes were isolated from hypericin-accumulating aerial tissues of several *Hypericum* representatives, except for the isolate 8RF1-3 from *H. humifusum* roots. Our previous results indicated that fungal endophytes are able to produce the same anthraquinones as the host plants [5, 7, 9]. The genome-mining of *Hypericum-*borne fungal endophytes led to the prediction of candidate biosynthetic gene clusters. In particular, non-ribosomal peptide synthetase (NRPS), PKS, terpene, indole, and hybrid (NRPS and PKS) clusters (Fig. 5) with diverse proportional representation in the analyzed genomes were detected. The number of BGCs in the studies fungal genomes differed. The isolate of *P. cucumerina* 2SF1-3 contained 24 BGCs, *S. apiospermum* 2RF1-5 37 and *C.* *subthermophilus* CS-A 42 BGCs. The predicted BGCs in *C.* *subthermophilus* with their core biosynthetic genes and putative products based on the homology with known clusters were summarized in Table S1. The highest overall number of BGCs in *D.* *eres* 17SF1-1 (109 BGCs) reflected the larger genome size compared to the other studied species. Forty-seven clusters found in *F.* *oxysporum* 8RF1-3 corresponded with the BGCs in 1LF1-1, with an exception of one NRPS-type cluster.

Comparative analysis of polyketide BGCs of the de novo assembled endophytic genomes and corresponding assemblies obtained from NCBI database showed that majority of predicted clusters have their counterparts in the reference genome with an exception of one additional BGC in the genome of *S.* *apiospermum* 2RF1-5 and two in *F.* *oxysporum*. On the other hand, only 35% of polyketide BGCs in *D.* *eres* 17SF1-1 showed similarity to the reference. Most of the PKS clusters in *C. subthermophilus* differed significantly from *T.* *terrestris*.

Polyketide BGCs comprised genes encoding PKS type I or type III as a core enzyme, additional tailoring enzymes, transport proteins, and fungal transcription factors. All the predicted iterative PKSs contained at least KS (β‐ketosynthase), AT (acyltransferase), and ACP (acyl carrier protein) or PP-binding domain (phosphopantetheine acyl carrier protein group). The most frequently observed domain organization of reducing type I PKSs was KS-AT-DH-MT-ER-KR-ACP (DH – dehydratase, MT – methyltransferase, ER – enoylreductase, KR – ketoreductase domain). AntiSMASH domain prediction algorithm also classified several ACP-lacking proteins as potential reducing PKSs. However, a deeper analysis of domain architecture indicated that these synthases are involved in the fatty acids biosynthesis.

**Fig. 6:**
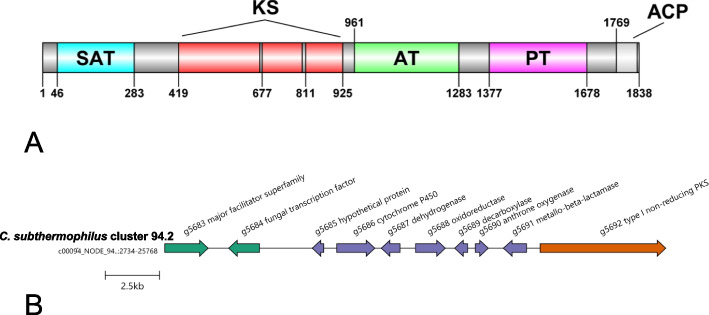
**A** Domain architecture of predicted non-reducing ‘endocrocin’ PKS in *C. subthermophilus*. Domain acronyms: SAT—starter unit:ACP transacylase, KS – β-ketosynthase, AT – acyltransferase, PT – product template, TE – thioesterase domain. **B** Candidate anthraquinone biosynthetic gene cluster in *C. subthermophilus*. Core biosynthetic gene is colored by tawny, tailoring genes by Chetwode blue and additional genes with non-catalytic function by green color

Non-reducing type I PKSs, involved in the biosynthesis of aromatic polyketides were less abundant than the reducing PKSs. The most prevalent domain structures of non-reducing PKSs were SAT-KS-AT-ACP-TE and SAT-KS-AT-PT-ACP(1-2x)-TE (SAT—starter unit:ACP transacylase, TE – thioesterase domain). It is assumed that fungal anthraquinone BGCs should comprise the non-reducing type I PKS (‘endocrocin PKS’ lacking TE domain) and discrete nearby β-lactamase-type thioesterase. Two non-reducing PKSs accompanied with β-lactamase in *D.* *eres* and one in *C. subthermophilus* genome were identified (Fig. 6A). Comparative analysis with previously identified fungal BGCs confirmed that the cluster 94.2 (the second cluster located in the scaffold No. 94) in *C. subthermophilus* genome contained putative homologs of *Escovopsis weberi* emodin biosynthetic genes: PKS (emoG), β-lactamase-type thioesterase (emoB), decarboxylase (emoH), anthrone oxidase (emoM), and major facilitator domain containing protein (MFS transporter) with the sequence similarity from 45 to 60%.

**Fig. 7:**
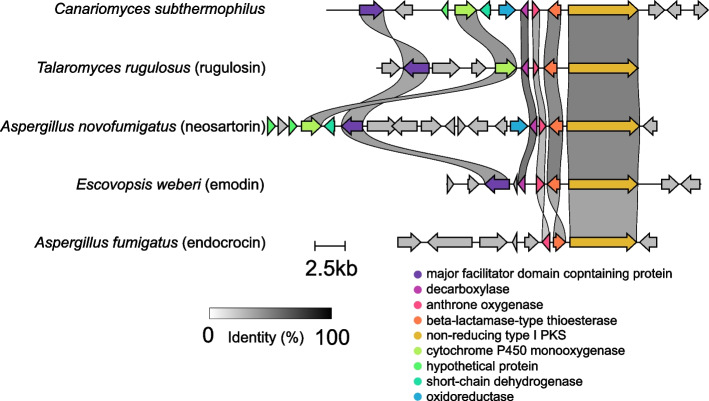
Comparison of candidate anthraquinone cluster from *C. subthermophilus* and identified BGCs of emodin type compounds in other fungi. Sequence similarity of predicted genes is depicted by grayscale

Predicted non-reducing PKS (g5692) contained SAT domain motif GXGXG typical for atrochrysone carboxylic acid synthases. Besides these genes, the genes functionally annotated as fungal-type transcription factor Zn2-Cys6 (g5684), hypothetical protein (g5685), cytochrome P450 monooxygenase (g5686), short chain dehydrogenase (g5687), and oxidoreductase (g5688) in the cluster 94.2 were identified (Fig. 6B). All the predicted genes and their functions are summarized in Table 2. Comparative analysis revealed a similarity with the fungal BGCs of dimeric polyketides, such as rugulosin from *Talaromyces rugulosus* W13939 or neosartorin from *Aspergillus novofumigatus* IBT (Fig. 7). It is presumed that dimerization step is catalyzed by predicted gene g5686 annotated as CYP450 monooxygenase sharing the sequence similarity of 52–53% with the predicted dimerization enzymes in *Talaromyces* and *Aspergillus* (rugG and nsrP). The predicted MFS transporter, CYP450 and enzymes catalyzing the steps to emodin or phenolic compounds with basic 9, 10-anthraquinone skeleton were identified as putative orthologs of skyrin biosynthetic genes (possible rugulosin precursor from *Talaromyces* sp.) with sequence similarity from 44 to 60%. The remaining genes g5685, g5687, g5688, and non-catalytical g5684 are presumably pathway-specific genes of novel candidate fungal bisanthraquinone gene cluster. The gene g5685 encodes a hypothetical conserved protein without any detected conserved domain. According to INTERPROSCAN analysis, the protein is a potential member of NTF2-like domain superfamily (nuclear transport factor 2) with the sequence similarity of the *A.* *novofumigatus* neosartorin isomerase nsrQ. The gene g5687 was annotated as short-chain dehydrogenase/reductase with a predicted NADP-binding active site and g5688 as a putative member of questin oxidase-like family. A conserved domain of unknown function (DUF4243) was detected in gene g5688 and functional analysis predicted oxidoreductase activity. These genes showed similarity with neosartorin reductase (nsrO, 67%) and oxidoreductase (nsrF, 59%).

**Table 2:** The gene composition of candidate anthraquinone BGC identified in *C. subthermophilus* genome. The gene functions were predicted by homology-based search against NCBI non-redundant protein database giving the identity of aligned sequences

Gene	Predicted function	Protein size (aa)	Putative homolog	Identity (%)
g5692	type I non-reducing PKS	1838	XP_003663601.1	81
g5691	metallo-beta-lactamase	327	KAH8777807.1	78
g5690	anthrone oxygenase	173	A0A4P8DK01.1	48
g5689	decarboxylase	152	TVY84600.1	78
g5688	oxidoreductase	457	GAQ08564.1	66
g5687	short chain dehydrogenase	294	KAF1349181.1	64
g5686	cytochrome P450 monooxygenase	525	SLM39059.1	58
g5685	hypothetical protein	149	XP_013322726.1	75
g5684	fungal transcription factor Zn2-Cys6	474	XP_003663605.1	53
g5683	major facilitator superfamily	583	KAH6839526.1	70

## Discussion

Plants and fungi are considered as the inexhaustible but underexplored source of valuable natural bioactive compounds. Various plant-colonizing endophytes are able to produce the same bioactive natural compounds as their hosts [10], and thus provide new biotechnological possibilities for large-scale production of valuable bioactive compounds. Fungal biosynthetic genes are often grouped into gene clusters (BGCs) comprising the genes encoding enzymes responsible for the formation and subsequent modification of metabolite scaffold, transport proteins and DNA-binding proteins. Computational approach is a powerful and rapidly evolving strategy for fungal genome-mining and discovery of novel biosynthetic genes candidates prior to experimental validation.

The representatives of the genus *Hypericum* produce several valuable phytochemicals. Hypericins are bioactive naphtodianthrones with a great biotechnological and therapeutical potential. The biosynthesis of hypericins that proceeds by the polyketide pathway through potential anthraquinone precursors emodin [11], emodin anthrone [12] or skyrin [5] has been generally accepted, but the successive steps are still unclear. Based on the knowledge that *Hypericum*-borne endophytic fungus, *C. subthermophilus* (formerly *Thielavia subthermophila*), is capable of emodin and hypericin synthesis in several subcultures of axenic culture [7], Henzelyová et al. [9] isolated and characterized more than 30 *Hypericum*-borne endophytes and some of them contained detectable amounts of emodin, emodin anthrone and bisanthraquinones. These endophytes along with *C. subthermophilus* were selected for genomic DNA sequencing and downstream bioinformatic analyses focused on the identification of candidate polyketide BGCs. As the genome of *C. subthermophilus* has not been sequenced yet, the assembly presented in this paper is the first reconstructed and characterized whole-genome sequence of this species. Taking into account GC content > 50%, large number of predicted complete genes and gene clusters together with relatively small genome size, it can be concluded that the genome of *C.* *subthermophilus* is highly compact. The annotation of *C.* *subthermophilus* mitogenome revealed *atp9* gene is absent. Several studies reported that fungal *atp9* gene was transferred into nuclear DNA [13]. Comparative analyses of the other studied *Hypericum*-borne endophytes with their reference genomes from NCBI database confirmed high level of synteny only in *F.* *oxysporum*, with two additional polyketide BGCs predicted in both isolates 1LF1-1 and 8RF1-3. Gottschalk et al. [14] observed similar differences of two genome assemblies of *F.* *avenaceum*. Pairwise comparisons of endophytic *D.* *eres, P. cucumerina* and *S. apiospermum* with the relevant reference strains revealed more frequent occurrence of indels, overall lower sequence similarity and diversity of BGCs repertoire, especially in *D. eres*. It is known that fungal isolates from distant geographic regions with diverse lifestyles may differ not only in the composition of BGCs but even in the chromosome sizes [15, 16].

Fungal PKS and hybrid NRPS-PKS BGCs represent a major class of biosynthetic clusters. Despite many polyketide BGCs have been identified in fungal genomes, linking of sophisticated bioinformatic prediction with metabolic pathways is still challenging. However, prediction of fungal biosynthetic genes may be complicated by branching of metabolic pathways and observed crosstalks between distant BGCs [17]. Most of the fungal anthraquinone derivatives are formed via emodin or chrysophanol precursor (reviewed by de Mattos-Shipley and Simpson [18]). The first steps of biosynthetic route (condensation of eight molecules of malonyl CoA and C6-C11 cyclization) are catalyzed by non-reducing iterative type I Group V PKS with the following features: i) lack of thioesterase domain (‘endocrocin-type’ PKS), ii) nearby discrete beta-metallo-lactamase-type thioesterase for the release step, iii) domain composition of SAT-KS-AT-PT-ACP, iv) loss of the active cystein site in SAT domain by C to G mutation in the motif GXGXG [19]. Based on the previous metabolomic analyses, such PKSs were expected in all studied isolates, except for *F.* *oxysporum* 8RF1-3. However, TE-lacking PKSs were identified only in *C. subthermophilus* and *D.* *eres*. Detailed analyses of domain architecture and multisequence alignments revealed that the only PKS that met all the mentioned criteria is the PKS from *C. subthermophilus* (cluster 94.2) showing similarity with endocrocin PKS from *A. fumigatus* [20]. The next steps of emodin biosynthesis are catalyzed by decarboxylase and anthrone oxidase [21]. The predicted gene arrangement in *C.* *subthermophilus* cluster 94.2 corresponded with the proposed emodin BGC from parasitic fungus *E. weberi* predicted by Heine et al. [22].

The basic anthraquinone skeleton is a key structure of many natural dimerized compounds, mainly bisanthraquinones and related xanthones (reviewed by Yuan et al. [23]). Dimerization step involves coupling of two precursor molecules by formation of C–C bond, which is assumed to be catalyzed by cytochrome P450 monooxygenase. Dimerization role of CYP450 was confirmed in the biosynthesis of bisanthraquinones (cladofulvin, skyrin) and xanthones (neosartorin) [24–26]. Predicted *C. subthermophilus* CYP450 showed higher sequence similarity to cytochrome P450 from *T. rugulosus* skyrin cluster (rugG) than CYPs from neosartorin and cladofulvin cluster. RugG is a substrate-promiscuous enzyme that accepts a broader spectrum of emodin-like substrate molecules and catalyzes C5-C5' dimerization to skyrin or bisanthraquinone intermediate air-oxidized to protohypericin [12].

The other candidate tailoring NADP-dependent short-chain dehydrogenase/reductase and predicted oxidoreductase showed similarity with reductase and ring-cleaving dioxygenase from BGC of neosartorin. However, functional classification of oxidoreductase into the questin oxidase-like protein family was ambiguous. Similarly, predicted hypothetical protein was identified as a member of very broad superfamily. Based on the sequence similarity with neosartorin isomerase, it can be presumed that this protein catalyzes interconversion of anthraquinone intermediates.

The alignment of *C. subthermophilus* cluster 94.2 and skyrin BGC from *T. rugulosus* showed that biosynthetic genes and additional transport protein are putative homologs. On the contrary, Jahn et al. [27] proposed skyrin BGC from *Cyanodermella asteris* composed of non-reducing PKS lacking SAT domain and containing TE domain, two short-chain dehydrogenases, scytalone dehydratase and flavin-binding monooxygenase with a different catalyzing mechanism than CYP450. Another PKS with similar domain layout KS-AT-PT-ACP-TE that catalyzes cyclic octaketide synthesis via atrochrysone, was recently identified in a macroscopic fungus *Cortinarius* sp. [28].

Different PKS domain layout and gene composition of BGCs indicates that anthraquinone biosynthetic routes evolved independently in various fungal taxons. The convergence of anthraquinone biosynthetic pathways is also expected between *Hypericum*-borne endophytes and hypericin-producing host plants. The suggested hypericin biosynthetic pathway in *Hypericum* spp. involves octaketide PKS type III [6, 29]. Compared to fungal multidomain type I PKSs, plant-type III PKSs comprise a single ketosynthase domain and thus presumably requires additional octaketide cyclase and thioesterase to form atrochrysone carboxylic acid further converted by decarboxylase and oxygenase to emodin. However, any candidate hypericin biosynthetic gene has not been validated yet.

## Conclusions

This work provides an insight into the genomes of *Hypericum*-borne endophytic fungi able to produce anthraquinones. The genomes of selected endophytes were sequenced and exploited by in silico analyses. The *C. subthermophilus* genome was completely sequenced and characterized for the first time. A potential anthraquinone BGC was discovered solely in the genomic sequence of *C. subthermophilus* using bioinformatic prediction and subsequent precise inspection of identified polyketide gene clusters. The cluster includes putative biosynthetic genes of emodin-type anthraquinone, cytochrome P450 monooxygenase with assumed dimerization role, other enzymes, fungal transcription factor and transport protein. Predicted gene cluster might be associated with the biosynthesis of bisanthraquinone further tailored by pathway-specific enzymes. The obtained computationally-guided results will be subjected to the experimental validation. The knowledge on genomic background of bisanthraquinone biosynthesis in endophytes is a key for understanding the anthraquinone biosynthesis in plants and for considering the simple eukaryotic system for prospective biotechnological production of hypericins and other potential host-plant derived anthraquinones with anticancer properties.

## Methods

### Fungal material and cultivation

Endophytic fungi isolated from *Hypericum* spp. were selected based on their ability to produce anthraquinones and bisanthraquinones innate to the host plants. The experimental group comprised two *Fusarium oxysporum* isolates varying in metabolite production, *Scedosporium apiospermum, Plectosphaerella cucumerina,* and *Diaporthe eres* which were collected from plants acclimated to outdoor conditions in the Botanical Garden of Pavol Jozef Šafárik University in Košice, Slovakia [9] (Table 3). The cultures were identified by molecular markers ITS rDNA and protein-coding gene region of tef1α. Moreover, fungi identified as *F. oxysporum* were classified to *F. oxysporum* species complex 33 based on the intron-rich portion of *tef1α* and maintained at the Institute of Biology and Ecology of Pavol Jozef Šafárik University in Košice, Slovakia, while *Canariomyces subthermophilus* (formerly *Thielavia subthermophila*) isolated from *Hypericum perforatum* of Himalayan provenance [7] was purchased from Leibnitz Institute DSMZ – German Collection of Microorganisms and Cell Cultures (accession number DSM 21024) and its authenticity was confirmed by ITS rDNA sequence. Fungal cultures stored at -80 °C in 20% (v/v) glycerol were transferred to potato dextrose agar (PDA, Difco) and cultivated in the dark at 26 °C with 7-day subculture interval. Before DNA extraction, fungal mycelia were transferred to potato dextrose broth (PDB, Difco) or malt extract broth (MEB) and cultivated for 7 days at 130 rpm in the dark at 26 °C.

**Table 3:** Endophytic fungi isolated from *Hypericum* species

Host plant	Plant tissue	Fungal species	Isolate code	Detected metabolites
*H. perforatum*	leaf	*Fusarium oxysporum*	1LF1-1	hypericin, pseudohypericin^a^
*H. humifusum*	root	*Fusarium oxysporum*	8RF1-3	n.d.^a^
*H. maculatum*	root	*Scedosporium apiospermum*	2RF1-5	emodin^a^
*H. maculatum*	stem	*Plectosphaerella cucumerina*	2SF1-3	emodin, emodin anthrone^a^
*H. tomentosum*	stem	*Diaporthe eres*	17SF1-1	emodin^a^
*H. perforatum*	stem	*Canariomyces subthermophilus*	CS-A	emodin, hypericin^b^

*n.d. *not detected

^a^Henzelyová et al*.* [9]

^b^Kusari et al*.* [7]

### Genomic DNA isolation and libraries preparation

Total DNA was extracted by NucleoSpin Soil Kit (MACHEREY–NAGEL) according to manufacturer’s instructions. Quality and concentration of total DNA was checked by spectrophotometer (Biotek Synergy HT) and fluorometer (Qubit 4.0) using dsDNA HS Assay kit. Swift 2S Turbo Flexible v2 DNA Library Kit with KAPA Unique Dual-Indexed Adapters was used to prepare libraries using 500 ng of genomic DNA per sample. Libraries were quantified by KAPA SYBR® FAST qPCR Kit on qTOWER^3^ Series real-time thermocycler (AnalytikJena) and fragments of prepared DNA libraries were examined by 1.8% TBE agarose gel electrophoresis. Final quality control was performed by High Sensitivity DNA Assay kit (Agilent Technologies) on Agilent 2100 Bioanalyzer.

### Whole-genome sequencing and assembly

The libraries were sequenced on Illumina platform NextSeq500 (CEITEC Masaryk University, Brno, Czech Republic) and MiSeq (Pavol Jozef Šafárik University in Košice, Slovakia) in paired-end mode with 2 × 150 bp read lengths and 100X coverage. After quality control by FastQC [30], the quality and adapter trimming was performed using Cutadapt V4.3 [31]. The trimmed reads were assembled de novo by SPAdes with different k-mer values (21, 33, 55, 77) [32]. The genes were predicted by stand-alone version of Augustus [33], a program based on the generalized Hidden Markov models. The prediction was performed using pre-trained model for *Fusarium graminearum*. The completeness of assembled genomes was evaluated by locally installed BUSCO (Benchmarking Universal Single-Copy Orthologs) software V5 based on the fungal database (fungi_odb10) [34], interactive web-based framework GenomeQC [35], and the assembly statistics were extracted using AGAT V1.2 [36]. The assembled genomes were aligned and compared to the reference genomes downloaded from NCBI database by MUMmer V4 [37] and D-GENIES [38]. The mitochondrial genomes were assembled from genomic data by GetOrganelle V1.7.7 [39] and annotated by MITOS V2.1.7 [40]. The circular map of *C.* *subthermophilus* mitogenome was drawn using Proksee [41].

### Functional annotation of gene and protein sequences

The predicted protein sequences were functionally annotated based on the orthology relationships using web version of EggNOG-mapper V2 [42]. The sequences were mapped against Eggnog database, KEGG pathways, Pfam, CAZy terms and COG functional categories [43–47]. The GO annotation results were processed by WEGO 2.0 [48]. The interspersed repeats and transposable elements were screened by RepeatMasker V4.0.9 [49] and de novo predicted by RepeatModeler V2.0.4 [50]. The genes encoding rRNA and tRNA were identified using barrnap V0.9 [51] and tRNAscan-SE V1.3.1 [52].

### Identification of orthologous gene families

The groups of orthologous genes (orthogroups) were inferred using OrthoFinder [53]. The species tree was constructed by STAG (Species Tree from All Genes) algorithm based on the sets of one-by-one orthologs in all species.

### Prediction of biosynthetic gene clusters

Prediction of biosynthetic gene clusters was performed via fungal version of antiSMASH V6.0 pipeline [54], using both genome assembly and feature annotation files with relaxed detection strictness. The pipeline comprised these successive steps:

i. analysis of PKS/NRPS (polyketide synthase/nonribosomal peptide synthetase) domain architecture,
ii. prediction of chemical structures,
iii. mapping on the database of gene clusters,
iv. analysis of the genomic sequences by the secondary metabolism Clusters of Orthologous Groups (smCOG).

The identified clusters were manually inspected and ambiguous gene functions were additionally verified by BLASTp or BLASTx against non-redundant NCBI protein database [55]. The multiple cluster alignments were performed by CAGECAT clinker [56]. The BGC GenBank files were obtained from MIBIG database [57] and NCBI. The PKS domain structure was visualized by DOG V2.0 [58].

### Supplementary Information

Supplementary Material 1.

## Data Availability

All data generated or analyzed during this study are publicly available in the NCBI database as BioProject No. PRJNA983573, BioSamples No. SAMN35731317—SAMN35731322.

## Abbreviations

ACP: Acyl carrier protein domain
AT: Acyltransferase domain
atp9: ATPase subunit 9
BGCs: Biosynthetic gene clusters
BUSCO: Benchmarking Universal Single-Copy Orthologs
CAZy: Carbohydrate-active enzymatic classes
COG: Classes of orthologous groups
CYP450: Cytochrome P450
DH: Dehydratase domain
ER: Enoylreductase domain
GO: Gene ontology
KEGG: Kyoto Encyclopedia of Genes and Genomes
KR: Ketoreductase domain
KS: β‐ketosynthase domain
MFS: Major facilitator transporter superfamily
MT: Methyltransferase domain
NADP: Nicotinamide-adenine-dinucleotide phosphate
NRPS: Non-ribosomal peptide synthase
nsrF: Oxidoreductase from neosartorin BGC
nsrO: Reductase from neosartorin BGC
nsrP: Cytochrome P450 from neosartorin BGC
nsrQ: Isomerase from neosartorin BGC
NTF2-like: Nuclear transport factor 2
PKS: Polyketide synthase
PT: Product template domain
rugG: Cytochrome P450 from rugulosin BGC
SAT: Starter unit:ACP transacylase domain
SM: Secondary metabolites
TE: Thioesterase domain
