# Supplementary material for: In silico prediction of polyketide biosynthetic gene clusters in the genomes of Hypericum-borne endophytic fungi
Source: BMC Genomics. 2024 Jun 3;25:555. doi: 10.1186/s12864-024-10475-z (PMC11149221; doi:10.1186/s12864-024-10475-z)
Supplement: Supplementary file 1 — Supplementary Material 1. [file 12864_2024_10475_MOESM1_ESM.docx]

**Supplementary materials**

***In silico* prediction of polyketide biosynthetic gene clusters in the genomes of *Hypericum*-borne endophytic fungi**

Linda Petijová*^1^, Jana Henzelyová^1^, Júlia Kuncová, Martina Matoušková, Eva Čellárová

Department of Genetics, Institute of Biology and Ecology, Faculty of Science, Pavol Jozef Šafárik University in Košice, Mánesova 23, 04154 Košice, Slovakia

* Corresponding author: [linda.petijova@upjs.sk](mailto:linda.petijova@upjs.sk)

^1^ These two authors contributed equally to this work


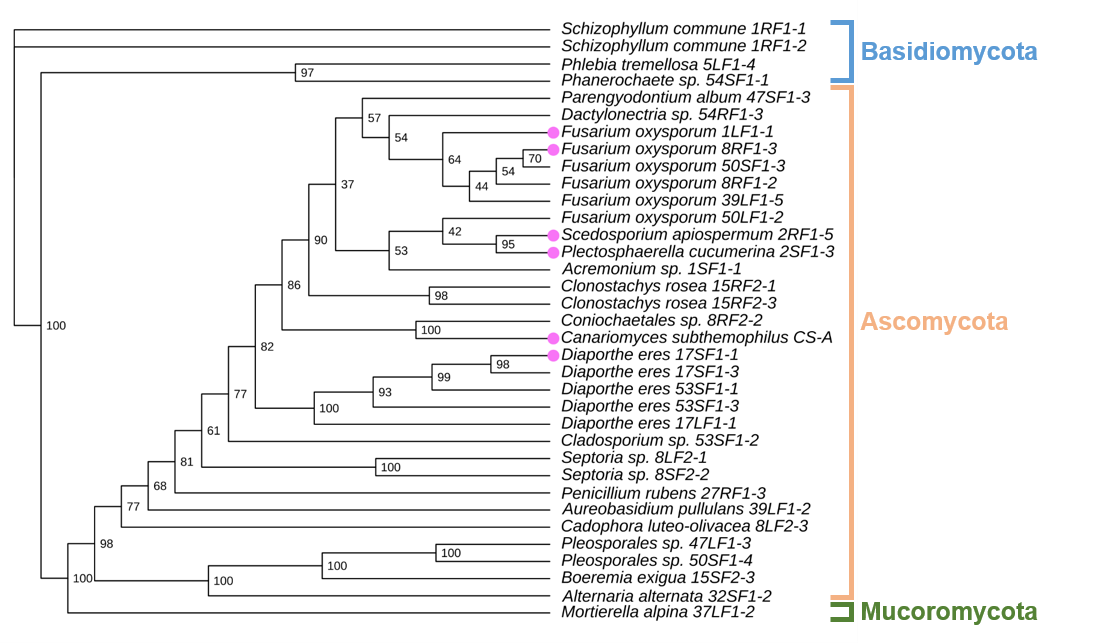


**Fig. S1** **The phylogenetic analysis of endophytic Hypericum-borne isolates.** The tree was constructed by maximum likelihood approach based on the ITS regions. The support bootstrap values were obtained from 1000 replicates.The studied isolates are marked by magenta circles. The isolates were collected and identified by Henzelyová et al. [8], C. subthemophilus was obtained from Leibnitz Institute DSMZ – German Collection of Microorganisms and Cell Cultures (accession number DSM 21024).


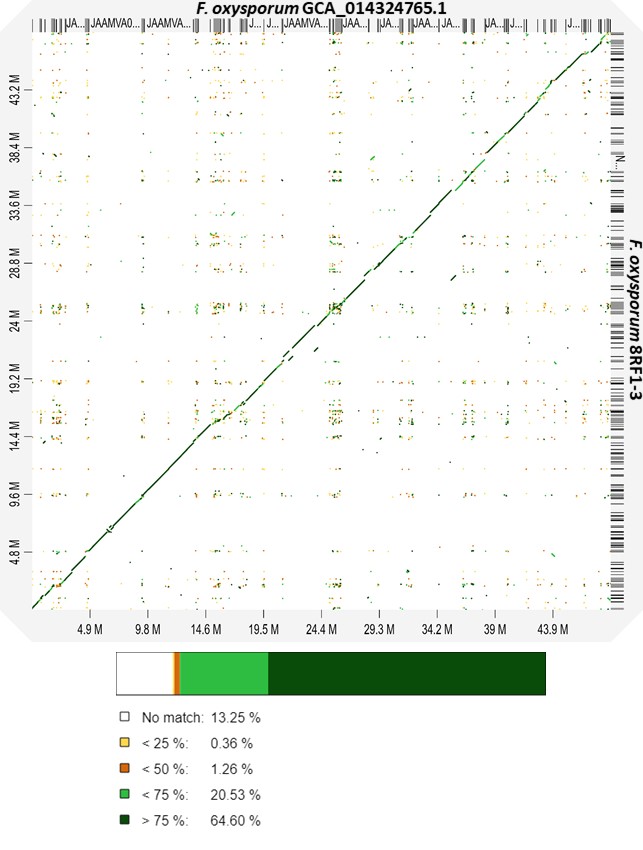


**Fig. S2** **The alignment of F. oxysporum GCA_014324765.1 reference genome and F. oxysporum 8RF1-3.** The alignments are colored according to the sequence identity.


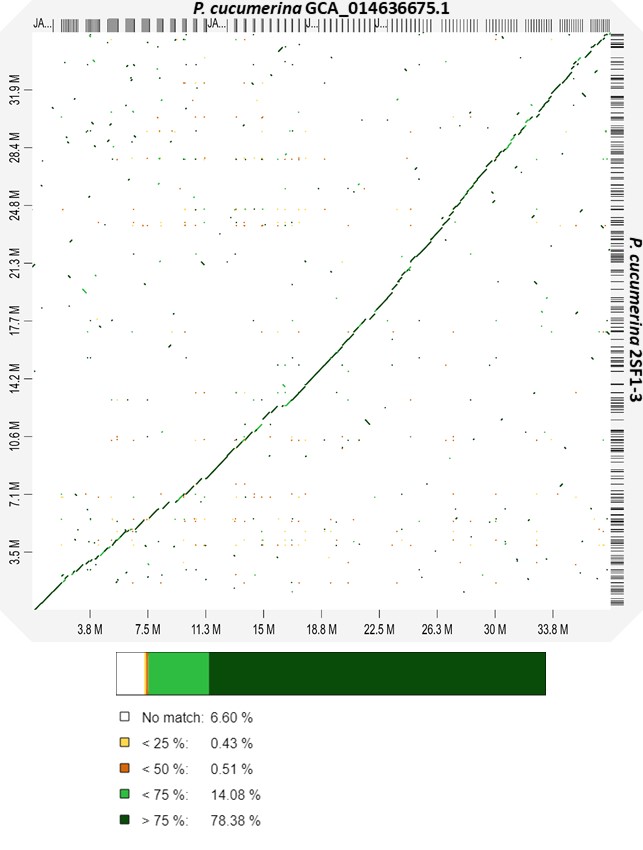


**Fig. S3** **The alignment of P. cucumerina GCA_014636675.1 reference genome and P. cucumerina 2SF1-3.** The alignments are colored according to the sequence identity.


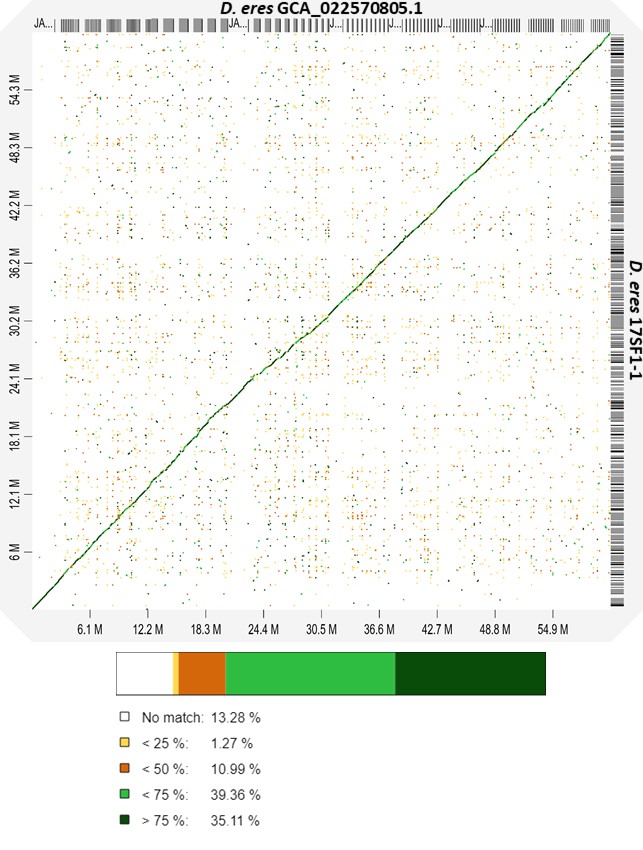


**Fig. S4** **The alignment of D. eres GCA_022570805.1 reference genome and D. eres 17SF1-1.** The alignments are colored according to the sequence identity.


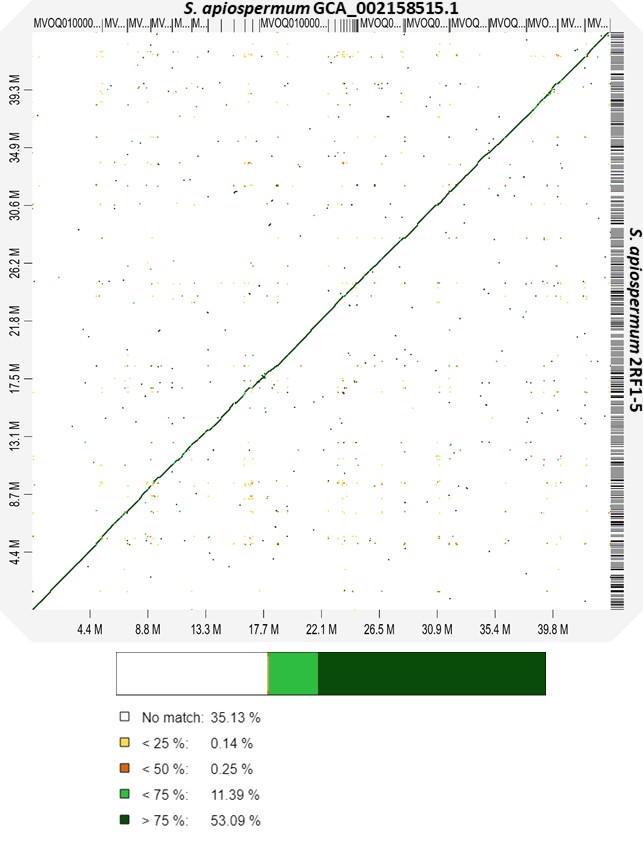


**Fig. S5** **The alignment of S. apiospermum GCA_002158515.1 reference genome and S. apiospermum 2RF1-5.** The alignments are colored according to the sequence identity.


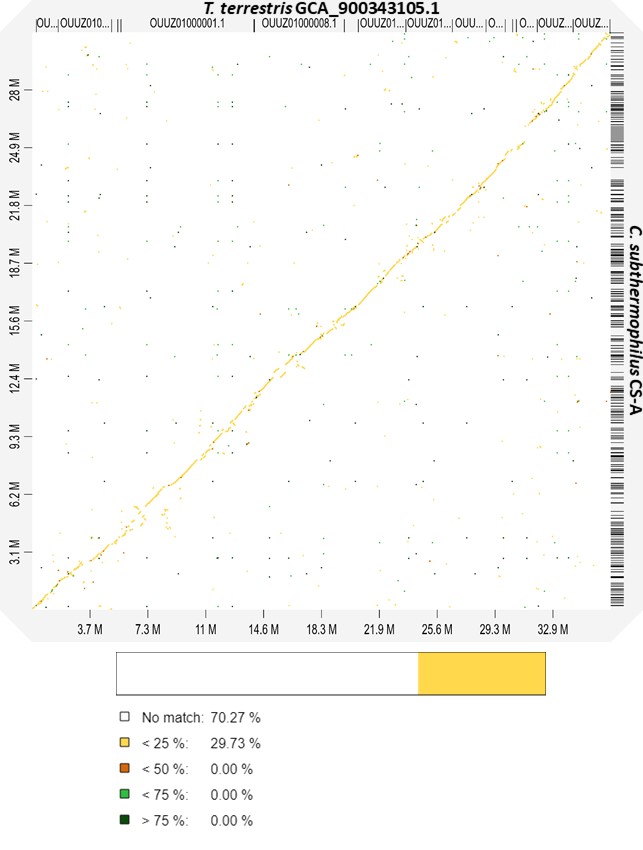


**Fig. S6** **The alignment of T. terrestris GCA_900343105.1** **reference genome and C. subthermophilus CS-A.** The alignments are colored according to the sequence identity.


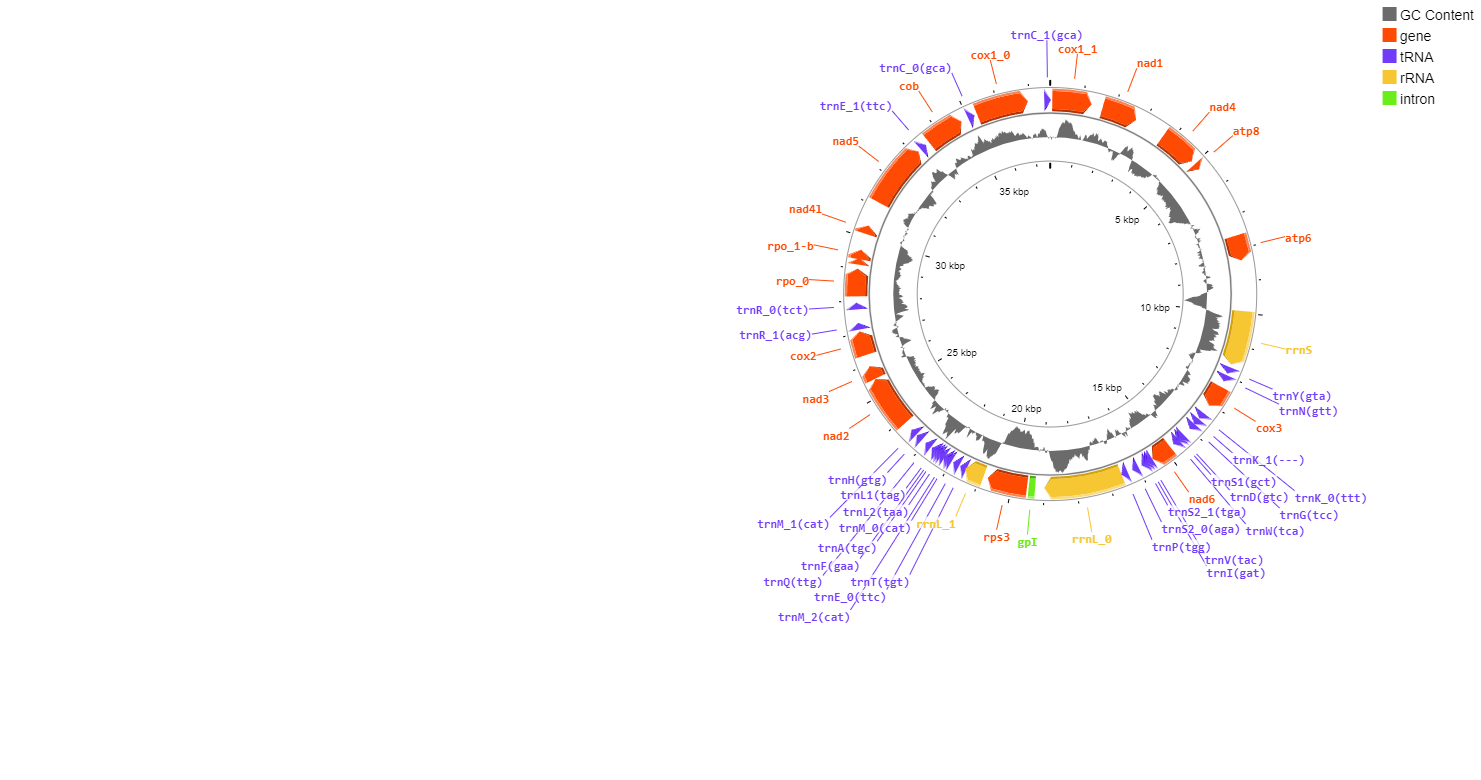


**Fig. S7 The circular map of C. subthermophilus CS-A mitochondrial genome.**


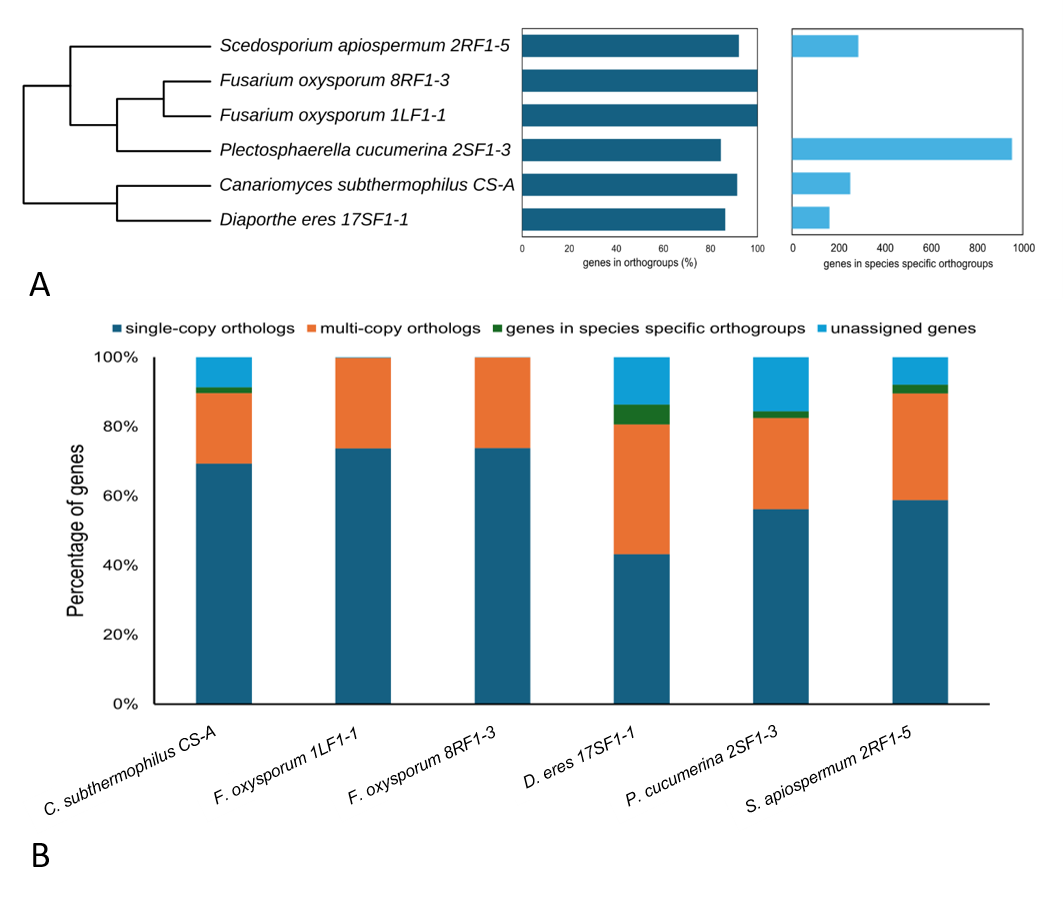


**Fig. S8 Phylogenetic analysis of studied fungal isolates (A).** The species phylogenetic tree was constructed by STAG algorithm, orthogroups were inferred by OrthoFinder. **More detailed classification of orthologous genes (B).**

**Table S1 Secondary metabolite BGCs identified in the genome of *C. subthermophilus* CS-A by antiSMASH.** The cluster products were predicted by comparison with known reference BGCs in MIBiG database.

| **BGC** | **BGC classification** | **Core biosynthetic gene** | **MIBiG comparison (compound/similarity score)** |
| --- | --- | --- | --- |
| 7.1 | type I PKS | reducing PKS | depudecin/0.65 |
| 13.1 | type I PKS | reducing PKS | betaenone/0.61 |
| 21.1 | terpene | geranylgeranyl pyrophosphate synthase | pimara-8(14),15-diene/0.50 |
| 22.1 | terpene | isoprenoid synthase | n.a. |
| 23.1 | terpene | farnesyl-diphosphate farnesyl transferase | squalestatin S1/0.62 |
| 30.1 | type I PKS | non-reducing PKS | alternariol/0.65 |
| 30.2 | type III PKS | polyketide synthase | alkyl-O-dihydrogeranyl-methoxyhydroquinone/0.47 |
| 34.1 | type I PKS | non-reducing PKS | monascorubin/0.70 |
| 41.1 | type I PKS | reducing PKS | mycophenolic acid/0.64 |
| 42.1 | type I PKS | reducing PKS | depudecin/0.66 |
| 47.1 | NRPS | nonribosomal peptide synthetase | xenematide/0.61 |
| 48.1 | type I PKS | reducing PKS | depudecin/0.55 |
| 53.1 | type I PKS, NRPS | hybrid PKS-NRPS | fusarin/0.67 |
| 57.1 | NRPS-like | nonribosomal peptide synthetase | livipeptin/0.53 |
| 59.1 | NRPS-like | nonribosomal peptide synthetase | fragin/0.54 |
| 59.2 | type I PKS | reducing PKS | alternapyrone/0.65 |
| 70.1 | type I PKS | reducing PKS | alternapyrone/0.65 |
| 71.1 | NRPS-like | nonribosomal peptide synthetase | fragin/0.53 |
| 73.1 | type I PKS | reducing PKS | alternapyrone/0.64 |
| 74.1 | type I PKS | reducing PKS | depudecin/0.59 |
| 79.1 | type I PKS | reducing PKS | alternapyrone/0.58 |
| 89.1 | type I PKS | reducing PKS | alternapyrone/0.62 |
| 94.1 | type I PKS | reducing PKS | betaenone/0.60 |
| 94.2 | type I PKS, NRPS | non-reducing PKS, nonribosomal peptide synthetase | emodin/0.71 |
| 98.1 | type I PKS | reducing PKS | depudecin/0.68 |
| 104.1 | NRPS, terpene | terpenoid synthase, nonribosomal peptide synthetase | basidioferrin/0.64 |
| 107.1 | terpene | terpenoid synthase | asperterpenoid A/0.47 |
| 128.1 | type I PKS | reducing PKS | betaenone/0.63 |
| 141.1 | type I PKS, NRPS | hybrid PKS-NRPS | fusarin/0.67 |
| 141.2 | type I PKS | non-reducing PKS | alternariol/0.66 |
| 151.1 | NRPS-like | nonribosomal peptide synthetase | fellutamide B/0.54 |
| 161.1 | type I PKS, NRPS-like | reducing PKS, nonribosomal peptide synthetase | LL-Z1272beta/0.80 |
| 163.1 | type I PKS, NRPS | reducing PKS, nonribosomal peptide synthetase | depudecin/0.66 |
| 168.1 | type I PKS, NRPS | hybrid PKS-NRPS | fusarin/0.67 |
| 171.1 | terpene | aristolochene synthase | PR-toxin/0.63 |
| 172.1 | type I PKS | reducing PKS | 6-methylsalicyclic acid/0.53 |
| 172.2 | terpene | terpenoid synthase | hirsutene/0.47 |
| 178.1 | type I PKS | reducing PKS | betaenone/0.61 |
| 187.1 | type I PKS | reducing PKS | mycophenolic acid/0.60 |
| 188.1 | NRPS | nonribosomal peptide synthetase | dimethylcoprogen/0.70 |
| 219.1 | type I PKS | non-reducing PKS | alternariol/0.63 |
| 243.1 | type I PKS | non-reducing PKS | 1,3,6,8-tetrahydroxynaphthalene/0.70 |
